# Effects of Formaldehyde on Lymphocyte Subsets and Cytokines in the Peripheral Blood of Exposed Workers

**DOI:** 10.1371/journal.pone.0104069

**Published:** 2014-08-26

**Authors:** Xiaowei Jia, Qiang Jia, Zhihu Zhang, Weimin Gao, Xianan Zhang, Yong Niu, Tao Meng, Bin Feng, Huawei Duan, Meng Ye, Yufei Dai, Zhongwei Jia, Yuxin Zheng

**Affiliations:** 1 Key laboratory of Chemical Safety and Health, National Institute of Occupational Health and Poison Control, Chinese Center for Disease Control and Prevention, Beijing, China; 2 Shandong Academy of Occupational Health and Occupational Medicine, Ji'nan, Shandong, China; 3 Department of Environmental Toxicology, The Institute of Environmental and Human Health, Texas Tech University, Lubbock, Texas, United States of America; 4 National Institute of Drug Dependence, Peking University, Beijing, China; Chang Gung University, Taiwan

## Abstract

Formaldehyde (FA) is a well-known irritant, and it is suggested to increase the risk of immune diseases and cancer. The present study aimed to evaluate the distribution of major lymphocyte subsets and cytokine expression profiles in the peripheral blood of FA-exposed workers. A total of 118 FA-exposed workers and 79 controls were enrolled in the study. High performance liquid chromatography, flow cytometry, and cytometric bead array were used to analyze FA in air sample and formic acid in urine, blood lymphocyte subpopulations, and serum cytokines, respectively. The FA-exposed workers were divided into low and high exposure groups according to their exposure levels. The results showed that both the low and high FA-exposed groups had a significant increase of formic acid in urine when compared to the controls. Both the low and high exposure groups had a significant increase in the percentage of B cells (CD19^+^) compared to the control group (*p*<0.01). A significant increase in the percentage of the natural killer (NK) cells (CD56^+^) was observed in the low exposure group compared to the control (*p* = 0.013). Moreover, the FA-exposed workers in both exposure groups showed a significant higher level of IL-10 but lower level of IL-8 than the control (*p*<0.01). Subjects in the high exposure group had a higher level of IL-4 but a lower level of IFN-γ than the control (*p*<0.05). Finally, there is a significant correlation between the levels of IL-10, IL-4, and IL-8 and formic acid (*p*<0.05). The findings from the present study may explain, at least in part, the association between FA exposure and immune diseases and cancer.

## Introduction

Formaldehyde (FA) is a ubiquitous pollutant in both outdoor and indoor air. It is also an important industrial chemical which has been widely used in construction, wood processing, carpeting, and so on. As a result, a large number of people are exposed to FA in the environment and/or workplace. FA is known to induce acute poisoning, respiratory irritation, as well as other immunotoxic effects and carcinogesis. It has been classified by the IARC as a human carcinogen that causes nasopharyngeal cancer and potentially leukemia [1]. Risk assessment of FA and leukemia has been challenging due to the inconsistencies in human and animal studies and the unknown mechanisms for leukemia induction. The relationship between FA exposure to respiratory and contact allergies has attracted considerable attention [2], [3]. Accumulating epidemiological evidence suggests that FA is a potential allergen for allergic contact dermatitis [4]–[7]. Moreover, it has been shown that the likelihood for the development of allergic asthma increases proportionately with indoor FA concentration [8], [9].

FA could affect the cell counts of different types of immune cells [10]. For instance, it was found that FA could increase the percentage of B cells, but decrease the percentage of total T cells (CD3^+^) and T-cytotoxic-suppressor cells (CD8^+^) in the blood of exposed workers, while T-helper-inducer cells (CD4^+^) remained unchanged [11]. Hosgood *et al.* recently found FA-exposed workers experienced decreased counts of natural killer (NK), CD4, and CD8 cells [10]. On the other hand, a previous study showed no difference in white blood cells, such as lymphocyte, or T-cells (CD4 and CD8) in subjects environmentally exposed to FA during an accidental spill [12]. The inconsistencies and limitations in these findings suggest that further study of the immunotoxic and hematological effects in populations with long-term FA exposure is important for understanding its adverse health effects.

Human CD4^+^ T cells are subdivided into T helper 1 (Th1) cells and T helper 2 (Th2) cells according to their distinct patterns of cytokine production. The former produces mainly IL-2 and IFN-γ, whereas the latter produces IL-4, IL-5, IL-6, and IL-10 [13]–[15]. A study by Ohtsuka *et al.* showed that after short-term FA inhalation, the levels of Th1-related cytokines (IFN-γ and IL-2) were significantly decreased, and Th2-related cytokines (IL-4 and IL-5) also tended to decrease in the nasal mucosa of Brown Norway rats [16]. Investigation of cytokine expression in peripheral blood will facilitate a better understanding of the differential function of T cell subsets in an immune response and carcinogenic effect. As of now, very few studies have evaluated the cytokine profiles in peripheral blood of FA-exposed workers; therefore, further study of the pathogenic role of cytokines is necessary.

In the present study, we aimed to examine the distribution of major lymphocyte subpopulations (T cells, B cells, and NK cells) in the peripheral blood of FA-exposed and unexposed workers. In addition, serum levels of the Th1 (IFN-γ)- and Th2 (IL-4 and IL-10)-related cytokines and the pro-inflammatory cytokines (IL-8 and TNF-α) in FA-exposed and unexposed workers were evaluated. Finally, the correlation between cytokine expression and FA exposure was assessed.

## Materials and Methods

### Study population

A total of 197 subjects were recruited for this study from East China in 2012, including 118 FA-exposed workers from a plywood factory and 79 workers from a food additive factory as non-exposure controls. The FA-exposed workers had been exposed to FA regularly for at least 6 months. The controls were recruited from a food additive factory producing monosodium glutamate, and they worked in quality inspection, packing, and storage. Exclusion criteria for both FA-exposed and control workers were a history of autoimmune diseases, infectious and immunocompromised diseases, and exposure to known mutagenic agents such as radiotherapy and chemotherapy within the preceding 3 months. All participants were interviewed by an occupational physician using a detailed questionnaire that included demographic information, educational level, smoking history, alcohol consumption, occupational history of exposure, and personal medical history. The participants provided venous blood and urine samples after a day's work. Major lymphocyte subsets were analyzed on the same day. Serum was separated within 4 h after collection, put into 1.5 mL tubes, stored at −80°C, and tested for the levels of cytokines. This study was approved by the Research Ethic Committee of the National Institute of Occupational Health and Poison Control, Chinese Center for Disease Control and Prevention, and written informed consent was obtained from each participant.

### Air sampling and FA exposure assessment

FA vapors in the plywood factory workshop came from the urea-formaldehyde glue used in the process of plywood production. FA-exposed workers were recruited from the following types of jobs: providing wood scraps, shaving wood, sanding boards, stacking boards, pressing ground wood scraps with glue at a high temperature, and making glue. The badges were connected to Gilian HFS-513A sampling pumps operating at a flow rate of 1.0 L/min for 8 h. Replications were done the next day. Two air-monitoring badges were set with a blank control in each of the six workplaces during production. FA concentrations of the samples were measured using high-performance liquid chromatography (HPLC) with UV (360 nm) detectors based on the NIOSH method 2016 (Issue 2). Exposed workers were classified into the low and high exposure groups based on their location and tasks within the plywood industry. The occupational exposure limitation (OEL) of FA in China is 0.5 mg/m^3^ (0.37 ppm). Workers making glue, pressing ground wood scraps with glue at a high temperature, stacking boards, sanding boards and post-processing, where the FA exposure level exceeds OEL, were assigned to the high exposure group, while workers from those shaving wood and providing wood scraps were assigned to the low exposure group.

### Flow cytometry measuring lymphocyte subsets

Cells were stained with monoclonal antibodies conjugated to fluorescein isothiocyanate (FITC), phycoerythrin (PE), or peridinin chlorophyll protein (PerCP) (Becton Dickinson, San Jose, CA, USA). Antibodies were used in the following combinations: panel 1, CD3-PerCP/CD4-FITC/CD8-PE; and panel 2, CD56-PE/CD19-FITC. Briefly, 100 µL of EDTA-preserved whole blood from each sample was added to individual test tubes containing pre-added monoclonal antibodies against CD3, CD4, CD8, CD19, and/or CD56 and incubated for 20 min at room temperature in the dark. After adding 600 µL formic acid (32 mM), the tube was vortexed for 30 second. The cells were washed twice with buffer solution (Na_2_CO_3_ 6 g/L, NaCl 14.5 g/L, and Na_2_SO_4_ 31.3 g/L) and re-suspended in 1% formaldehyde. Cell acquisition was performed using a BD FACSArial II, followed by analysis using FACS DIVA software (Becton Dickinson).

### Cytokine measurement

Serum cytokine concentrations, including IL-4, IL-8, IL-10, IFN-γ, and TNF-α, were measured using the BD Cytometric Bead Array Human Soluble Protein Flex Set system (Becton–Dickinson). Fifty microliters of each serum sample were used. Fluorescence intensity (FI) from the immunoassay was acquired and analyzed using the BD LSR II system and BD FCAP Array Software (Becton Dickinson).

### Formic acid measurement

Urine samples were collected from the participants by polyethylene plastic bottles after a day's work. The urine was separated within 4 h after collection, stored at −80°C, and tested for the levels of formic acid within one week. A total of 200 µL of urine from each sample was added to individual test tubes containing pre-added 200 µL of phosphate buffer solution (PH = 7.6). After adding 1.0 mL of 1-bromo methyl-5-phenyl fluoride solution (20 µg/µL), the tube was vortexed for 1 min and incubated for 1 h at 60°C. The upper organic phase was extracted by n-hexane, and filtered to autosampler vial (membrane pore size is 0.45 µm). Thirty µL of treated sample was detected for the level of formic acid by HPLC (Agilent, USA). The final formic acid concentration was adjusted by urine creatinine.

### Chemicals and antibodies

Dulbecco's phosphate buffered solution (D-PBS) was obtained from Gibco BRL (Paisley, Scotland, UK). All other chemicals were from Beijing Chemical Works (Beijing, China) and were with analytical grade or the highest commercial grade available. All monoclonal antibodies conjugated to fluorescein isothiocyanate (FITC), phycoerythrin (PE), or peridinin chlorophyll protein (PerCP), were from Becton Dickinson (BD). 1-bromo methyl-5-phenyl fluoride was from Acros Organics (Acros Organics, Belgium)

### Statistical analysis

Comparisons of baseline demographic characteristics of subjects were conducted by one-way analysis of variance (ANOVA) test or Pearson χ^2^. The differences of cytokines among the high exposure group, the low exposure group, and controls were compared by a nonparametric Mann-Whitney U-test. The two-tailed, nonparametric Spearman method was used to assess correlations between the cytokine concentrations and the level of formic acid in urine. All statistical analyses were performed using STATA 10.0 software. Differences were considered to be statistically significant if p<0.05.

## Results

### FA concentrations

The low and high exposure groups had an average FA exposure level of 0.15 ppm (range: 0.07 to 0.23 ppm) and 0.63 ppm (range: 0.36 to 1.53 ppm), respectively (Table 1). The concentrations of FA were much higher in the two exposed groups than in the control group. The level of FA in the high exposure group exceeded the recommended OEL in China (0.37 ppm). The levels of FA in the blank control and the control factory were under the limit of detection (0.008 ppm). None of the recruited workers had been recently exposed to FA in their home environments, according to the survey of house redecoration.

**Table 1:** The FA exposure levels in FA-exposed workers and controls.

Groups	Number of workers	Number of air samples	FA concentrations (ppm)	
			mean	range
High exposure	70	16	0.63	0.36–1.53
Low exposure	48	8	0.15	0.07–0.19
Controls	79	4	<0.008^a^	

a The limit of detection (0.008 ppm) was used to present the FA level in the control industry.

### The demographic characteristics of the study population

The demographic data of the study subjects are summarized in Table 2. The distribution of age, gender, Body Mass Index (BMI), and the history of smoking and alcohol usage were not significantly different among the three study groups. No significant difference in years of employment was observed between the two FA-exposed groups. The averages of age were 36.04±1.17 and 35.80±0.91 years in the low exposure group and the high exposure group, respectively. Males accounted for 72.92% and 75.71% in the low exposure group and the high exposure group, respectively.

**Table 2:** Demographic data of FA-exposed workers and controls.

Variables	Control group (n = 79)	Low exposure group (n = 48)	High exposure group (n = 70)	P	P	P
				C vs L	C vs H	L vs H
Age (years, mean±SE)	36.84±1.01	36.04±1.17	35.8±0.91	0.60^a^	0.44^a^	0.88^a^
Sex (male, *n* (%))	62 (78.48)	35 (72.92)	53 (75.71)	0.47^b^	0.69^b^	0.73^b^
BMI (mean±SE)	24.59±0.42	24.03±0.44	24.06±0.34	0.35^a^	0.32^a^	0.96^a^
Current smokers (yes, n(%))	26 (32.91)	12 (25.00)	25 (35.71)	0.35^b^	0.72^b^	0.22^b^
Alcohol user (yes, n(%))	44 (55.70)	28 (58.33)	41 (58.57)	0.77^b^	0.72^b^	0.98^b^
FA exposure history (years, mean±SE)	--	3.89±0.41	4.29±0.35	--	--	0.46^c^

a one-way ANOVA for difference between any two groups.

b χ^2^tests for differences between any two groups.

c one-way ANOVA for difference between the low and high exposure groups.

“C vs L” means “control vs low exposure group”, “C vs H” means “control vs high exposure group” and “L vs H” means “low exposure group vs high exposure group”.

### The concentrations of formic acid in study participants

**Figure 1:**
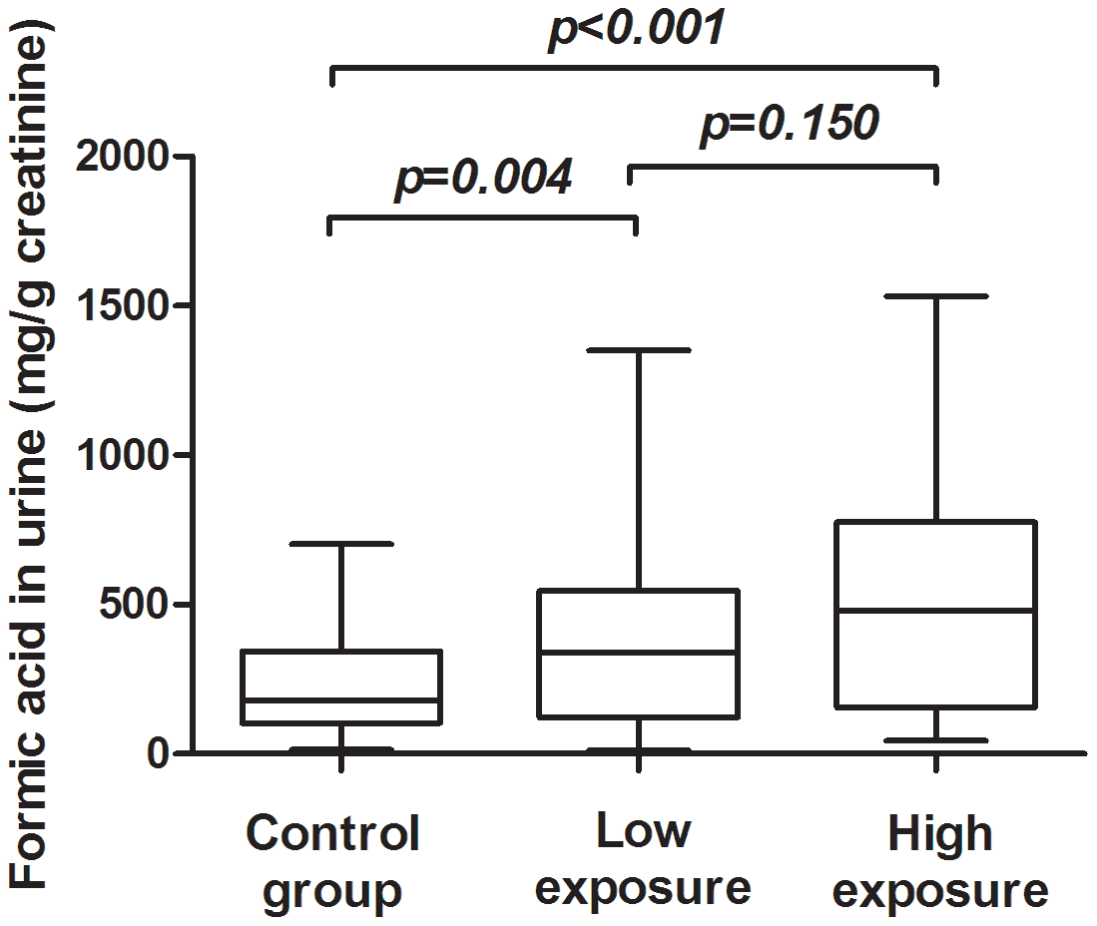
The level of formic acid in FA-exposed workers (n = 48 in the low exposure group and n = 70 in the high exposure group) and the control (n = 79). Sample was detected for the level of formic acid by high performance liquid chromatograph (HPLC). The final formic acid concentration was adjusted by urine creatinine.

As shown in Fig. 1, the two exposed subgroups had a significant increase of formic acid compared to the control group (*p*<0.01). However, no significant difference in the concentration of formic acid was observed between the low exposure group and the high exposure group.

### Phenotypic characterization of lymphocytes

**Figure 2:**
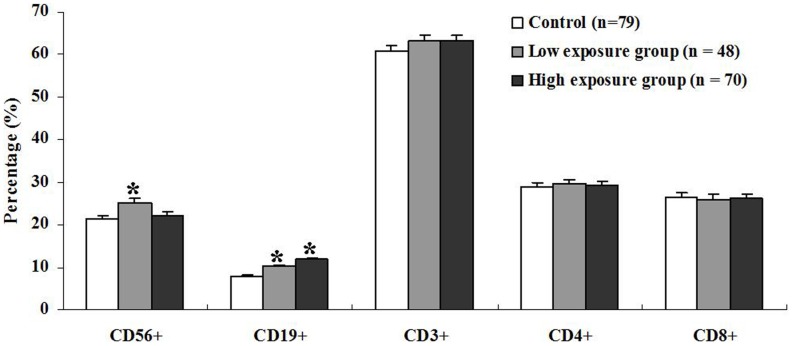
The distribution of major lymphocyte subsets in FA-exposed workers (n = 48 in the low exposure group and n = 70 in the high exposure group) and the control (n = 79). Percentage of different lymphocytes was presented in the three groups. Aliquots of EDTA whole blood were stained with fluorescence labeled antibodies to the CD3^+^, CD4^+^, CD8^+^, CD19^+^ and/or CD56^+^ lymphocytes. Values represent mean±SE; * p<0.05 comparing with control.

Venous blood samples from the workers in the three groups were analyzed by flow cytometry for enumerating different lymphocyte subsets. The tendency of an increase in the percentage of B cells (CD19^+^) was found with an increase level of FA exposure (Fig. 2). Both the low and high exposure groups had a significant increase in the percentage of B cells compared to the control group (*p*<0.01), and the highest percentage of B cells was observed in the high exposure group. The percentage of NK cells (CD56^+^) was significantly higher in the low exposure group than in the control (*p* = 0.013). However, no significant increase in the percentage of NK cells was observed in the high exposure group compared to the control group (Fig. 2). The percentages of total T cells (CD3^+^), T-cytotoxic-suppressor cells (CD8^+^), and T-helper-inducer cell (CD4^+^) were not significantly different between the control and the FA-exposed groups (Fig. 2).

### The levels of serum cytokines

**Figure 3:**
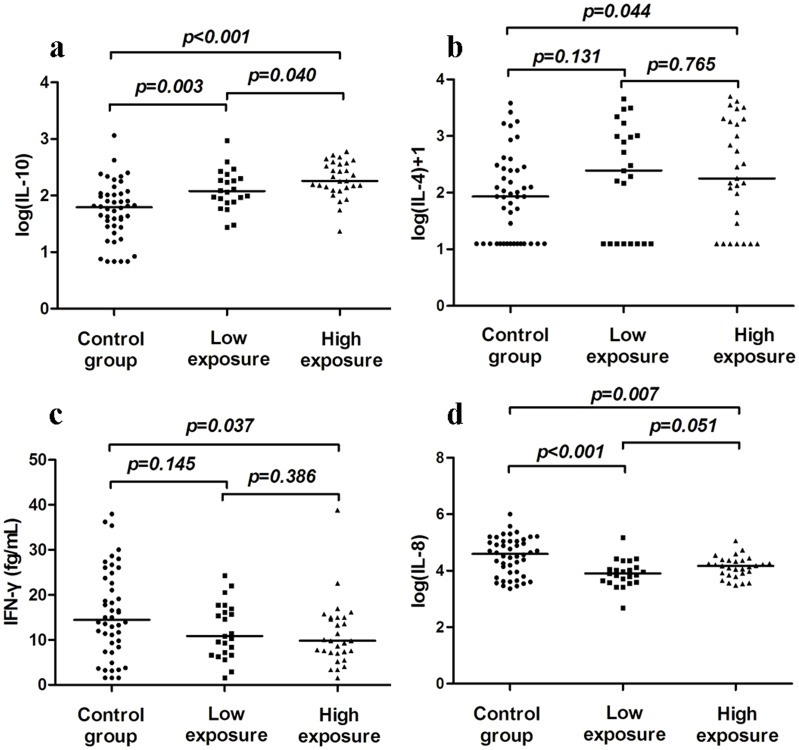
Comparison of serum cytokine levels between FA-exposed workers (n = 23 in the low exposure group and n = 29 in the high exposure group) and the non-exposed controls (n = 46). Serum levels of IL-10 (a), IL-4 (b), IFN-γ (c), and IL-8 (d) were measured by multiplexed bead immunoassay. Mann–Whitney U-test was performed to compare FA-exposed workers with the control.

**Figure 4:**
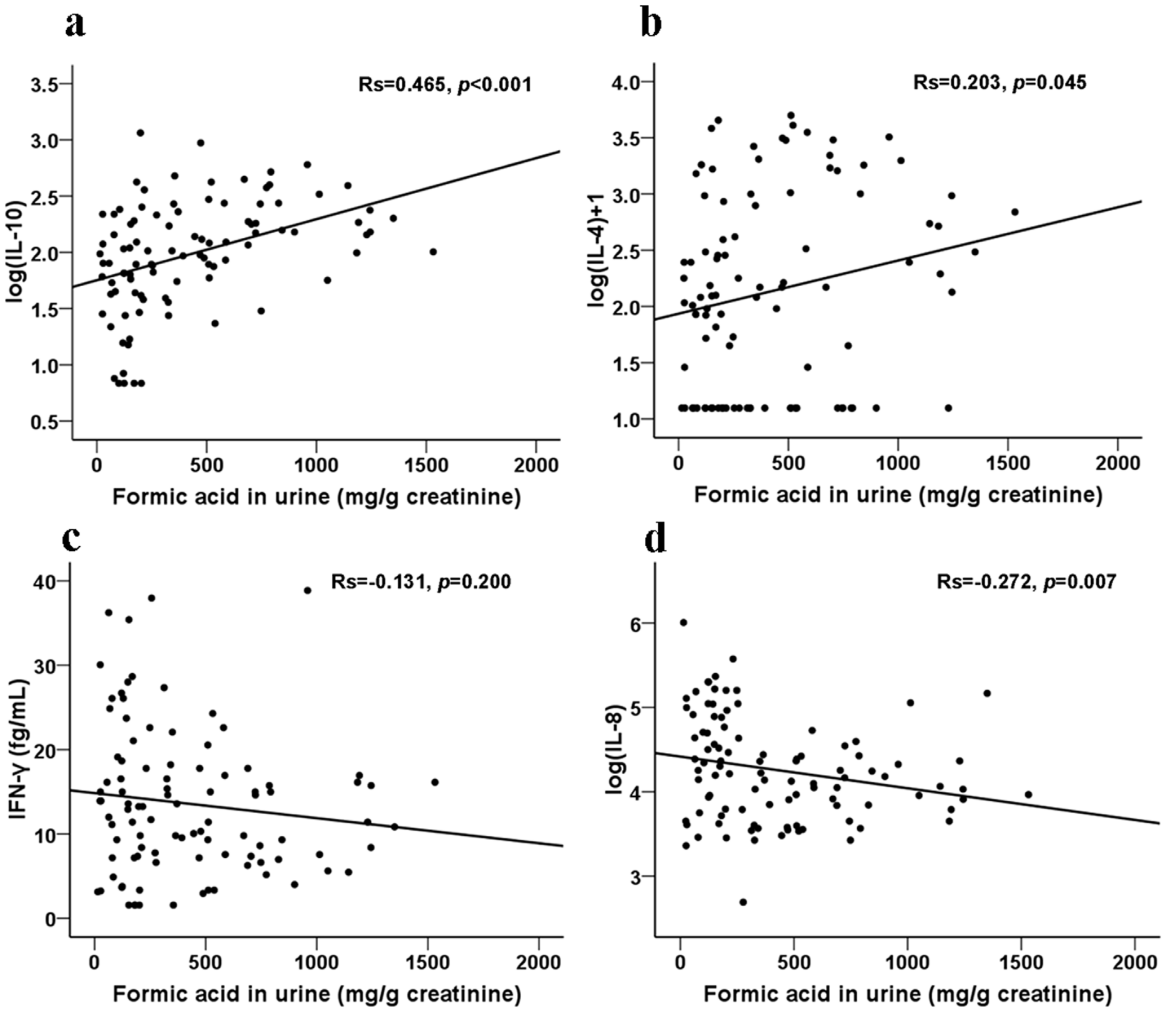
Correlation between formic acid and serum cytokine levels. Correlation between formic acid and IL-10 (a), IL-4 (b), IFN-γ (c), or IL-8 (d).

The 52 FA-exposed workers (29 workers from the high exposure group and 23 workers from the low exposure group) and 46 non-exposed controls were randomly selected to assess the concentrations of IFN-γ, IL-4, IL-10, IL-8, and TNF-α in serum samples. The distribution of age, gender, BMI, and history of smoking and alcohol usage were not significantly different between any two groups. If the concentrations of cytokines from individual sample were lower than the detection limit of the analysis, one half of the low detection limit was substituted for the level(s) of cytokine(s) for that sample [17]. As shown in Fig. 3a, the FA-exposed workers showed significantly higher levels of IL-10 in the low and high exposure groups than the control (low exposure *vs.* control, *p* = 0.003; and high exposure *vs.* control, *p*<0.001). The level of IL-10 was also significantly higher in the high exposure group than in the low exposure group (*p* = 0.04). Subjects in the high exposure group had a higher level of IL-4 than the control group (*p* = 0.044) (Fig. 3b). However, with respect to IFN-γ, we observed a significantly lower level in the high exposure group compared to the control (*p* = 0.037) (Fig. 3c). The levels of IL-8 were significantly lower in both the low exposure group and the high exposure group than in the control group (low exposure *vs.* control, *p*<0.001; and high exposure *vs.* control, *p* = 0.007; Fig. 3d). TNF-α levels showed no significant difference among the three groups (data not shown). Correlation analyses were performed to determine the relationship between the serum levels of cytokines and formic acid (Fig. 4). The levels of serum IL-10 (rs = 0.465, *p*<0.001), IL-4 (rs = 0.203, *p* = 0.045), and IL-8 (rs = -0.272, *p* = 0.007) were correlated with formic acid. No significant correlation was observed between formic acid and IFN-γ.

## Discussion

The epidemiological literature on the relationship between FA exposure and immune disease and cancer is extensive, and many studies suggest an association between them. Some studies have attempted to evaluate the impact of FA on different types of blood cells and soluble mediators such as cytokines and chemokines [10], [12], [16], [18], [19]. These studies were inconsistent and had limited information on lymphocyte composition and profiles of the Th1 and Th2 cytokines in FA-exposed workers. Hence, more detailed and comprehensive studies on the immunotoxicity and hematotoxicity of FA in exposed populations are necessary. In the present study, we described the lymphocyte distribution and cytokine expression profiles in the peripheral blood of FA-exposed workers. We demonstrated that the percentage of B cells increased with the increasing level of FA exposure. The percentage of NK cells was high in the low exposure group. Our results also revealed that FA exposure can promote Th2-skewed immune responses, as reflected by the high expression of IL-10 and IL-4, and the low expression of IFN-γ.

It is well known that FA is metabolized to formic acid in the body, and then excreted through the urine. Theoretically, exposure to FA could create a shift in the formic acid levels in the urine. The suitability of measuring formic acid in urine as a parameter for the biological monitoring of inhalational exposure to FA is still controversial [20], [21]. In our study, we found a significant increase in formic acid in the urine of plywood workers exposed to FA compared with the control.

Lymphocyte analyses in blood are widely used to evaluate the immune status in patients with various diseases. In our study, there were no significant differences in CD4^+^, CD8^+^, and CD3^+^ T cell subsets in the blood of FA-exposed workers *vs.* non-exposed controls. Similar to our results, a study by Madison *et al.* found that people environmentally exposed to FA during an accidental spill showed no difference in white blood cells, lymphocytes, or T-cells (CD4 and CD8) [12]. However, these findings are not corroborated by some studies that show lower proportions of these cells in FA-exposed worker than in controls [10], [11]. For instance, the study by Ye *et al.* showed that the decreased ratio of T lymphocytes, especially the decrease of the CD8^+^ subset, was observed in FA-exposed workers [11]. Similar results were found in FA-exposed students by the same research group [22]. Since these studies took place in varied settings with different co-exposures, it is likely that the uncontrolled confounders could explain some of these results.

There is growing evidence that NK cells play an important role in anti-tumor and antiviral immune responses. In the present study, the proportions of NK cells were significantly higher in the low exposure group, but not the high exposure group when compared with the control. Our results are consistent with the findings from the study by Aydin *et al.* [23]. On the contrary, previous studies have shown reduced ratios of NK cells in FA-exposed workers and mice compared with controls [10], [24]. Moreover, a recent study also revealed that FA could induce NK cell death in a concentration-dependent manner [25]. Altogether, the discrepant findings of NK cells after FA exposure might result from different concentrations of FA.

Our findings of higher proportions of B cells in the blood is in agreement with previous studies that demonstrate increased B cells in both FA-exposed workers and students [11], [22]. The B lymphocytes are the effectors of humoral immunity. Elevated B cell counts in subjects with long-term exposure to FA were previously reported by Thrasher *et al.* [26], and an activated immune system was shown in another report by Madison *et al.* [12]. Though the mechanisms by which FA affects blood lymphocytes and/or lymphocyte subsets are still unknown, the results from previous studies and the findings in the present study demonstrated altered lymphocytes in long-term FA-exposed workers. As the healthy immune system recognizes and destroys nascent transformed cancer cells, the observed dysregulation of immune function could play an important role in FA-induced cancer.

Th1 cells are considered the mediators of cellular immunity, while Th2 cells are the mediators of humoral immunity [27]. IL-10 is a potent anti-inflammatory cytokine that suppresses the secretion of proinflammatory cytokines [28], allergen-induced airway inflammation, and non-specific airway responsiveness [29], [30]. For instance, several T cell subsets in humans express IL-10 and utilize it to suppress the expression of IFN–γ to skew a Th2 response [31]. In addition, IL-10 showed regulatory effects on Th1 responses [32]. Our finding of the increased serum level of IL-10 in FA-exposed workers concurs with a previous report that there is an increased IL-10 release in human respiratory epithelium after acute exposure to FA [33]. IL-4 is of critical importance to the differentiation of Th2 cells. It is also a critical factor for the development of type 2 immune responses [34], [35]. IL-4 directly induces the differentiation of T cells into the committed effector Th2 lymphocytes [36]. Our findings of the increased serum levels of IL-4 in FA-exposed workers concur with previous reports that there was an increased IL-4 release in FA-treated mice [18], [19].

IL-8, besides its central role in inflammation, has biological functions including T cell chemotaxis [37] and hematopoiesis [38]. In this study, there was a lower expression of IL-8 in FA-exposed workers than in the control. Enhanced IL-8 release was observed in human lung cells exposed to FA [39], whereas FA inhibited IL-8 production was also reported in bronchial epithelial cells [40]. IFN-γ, a representative cytokine of Th1 cells [41], can enhance MHC-II expression and promote antigen presentation and activation on macrophages to start the body's immune response [42]. Our findings of low expression of IFN-γ in the blood are in line with previous studies that demonstrate selectively suppressed IFN–γ expression following exposure to FA or its mixture (with benzene, toluene, and xylene) [18], [43].

The regulation of Th1/Th2 cytokines determines the outcome of the immune response and the development of diseases [41], [44]–[46]. During the immune response, B-cells are significantly activated by the cytokines produced by Th2 T-cells, specifically, IL-4, IL-5, and IL-10 [47]. Our findings of a dose-dependent higher proportion of B cells in blood and increased serum levels of IL-4 and IL-10 further imply the potential importance of humoral immunity in FA-exposed workers. There is growing evidence that FA can cause asthma in human and mice who experience long-term FA exposure [48], [49]. The rise in IL-4 and IL-10 levels accompanied by the decrease of IFN-γ levels in our findings may contribute, at least partly, to allergic airway inflammation.

Most chronic diseases, including cancers, are caused by a dysregulated inflammatory response. It was well known that inflammation microenvironments play critical roles in tumor development and progression. Tumor development and growth are driven in many cases by inflammatory cells, which produce cytokines that subsequently stimulate the growth and survival of malignant cells [50]. Excessive production of IL-8 may be involved in the pathogenesis of several types of inflammatory reactions. Potent inhibitors of IL-8 production include dexamethasone, IL-4, and IL-10 [51]–[53]. Therefore, in the present study, the Th2 response characterized by a rise in IL-10 and IL-4 levels may partly contribute to the suppression of IL-8. The suppression of IL-8 production may be beneficial to the worker in controlling the inflammatory reactions.

Our population study has some limitations. Although smoking and house redecoration were assessed as environmental FA exposures in this study, exposure from diet was not considered. Therefore, we cannot exclude the possibility that our results might be influenced by these potentially confounding factors. Moreover, determination of the personal FA exposure levels of workers may provide more accurate evaluation of the dose-response relationship between FA exposure and immune response than the estimation of exposure levels based on workplaces or job types.

To summarize, besides the imbalance of Th cytokine expression, the significantly higher ratio of B cells in peripheral blood confirms a predominance of humoral immunity as an immune response in FA-exposed workers. Moreover, the abnormal expression of Th cytokines and proinflammatory cytokines presented here may account, at least in part, for the association between chronic FA exposure and immune diseases and cancer.
